# CMR at 3.0T in routine clinical practice - tips and tricks to optimise image quality and enhance patient flow

**DOI:** 10.1186/1532-429X-17-S1-T10

**Published:** 2015-02-03

**Authors:** Joanne Wormleighton, Kelly Parke, Joanne Carlton, Odette Thraves, Gerry P McCann

**Affiliations:** 1 Imaging, Glenfield Hospital, University Hospital of Leicester NHS Trust, Leicester, UK; 2 Cardiovascular Sciences, Glenfield Hospital, University Hospitals of Leicester, Leicester, UK

## Background

Cardiac magnetic resonance (CMR) imaging at 3.0T results in improved signal-to-noise ratio (SNR), however additional challenges arise that affect image quality. Here, we describe frequently encountered artefacts at 3.0T and how to optimise scanning techniques to provide good quality cardiac images.

## Methods

Glenfield Hospital (Leicester, UK) performs ~ 2500 CMR scans annually, 700 at 3.0T. These include complex congenital and stress perfusion scans as well as a diverse portfolio of research studies. Image optimisation methods (described below) can prevent or limit general and specific artefacts seen at 3T, improving image quality and patient through-put.

## Results

### ECG Gating

Retrospective as default

Arrhythmia detection for occasional ectopics

Prospective for frequent ectopics or atrial fibrillation, using short acquisition window

Finger-pulse oximiter when ECG fails

Real-time for poor ECG and poor breath-holding

### Alter Breath-hold duration

Increasing parallel-imaging factors (up to x 4 routinely) will reduce breath-hold with minimal impact on SSFP quality

Reduce spatial resolution - small reductions in phase resolution will significantly reduce breath-hold

Increasing the echo train/segments shorten breath-hold, with slight decrease in temporal resolution

Increase the number of averages(3-5) for free-breathing cine images in sedated/deaf subjects

Utilise increased SNR to acquire 2 slices per breath-hold in good breath holders to shorten exam time

### Sequence-specific cardiac shim

CMR at 3.0T requires careful, targeted shimming to significantly improve image quality for SSFP cine, T1-mapping and myocardial nulling on late gadolinium enhanced images.

### Off resonance artefacts

**Figure 1:**
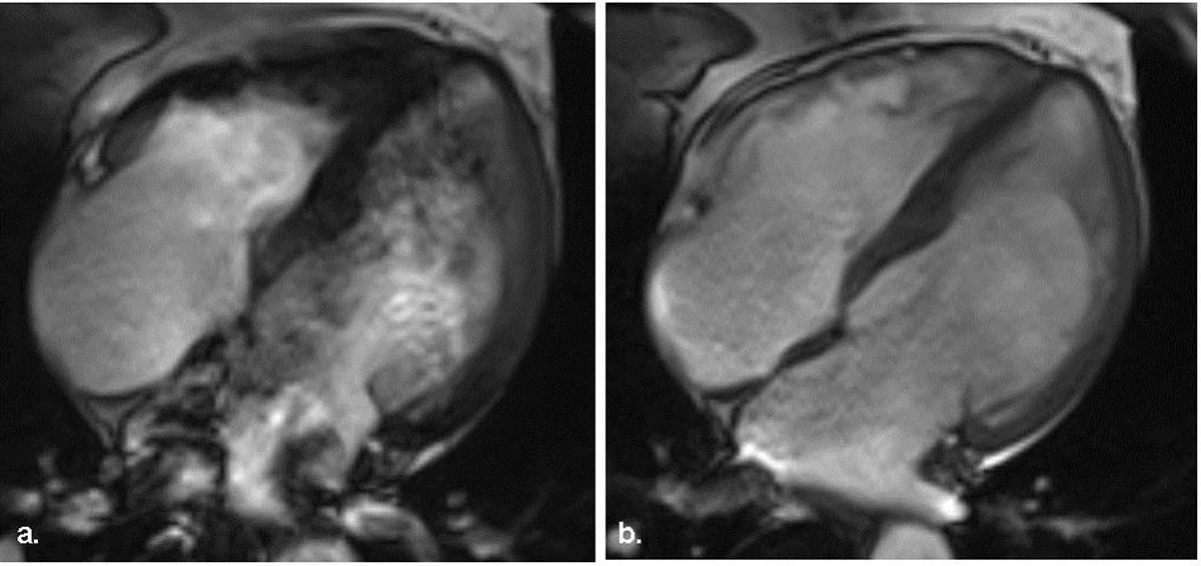
(a) A 4 chamber still of an SSFP cine with no adjustment to the frequency offset showing off resonance artifact. (b) An adustment of +50Hz to the frequency minimised the off resonance artifact.

Frequently occur at 3.0T on SSFP images; adjusting the radiofrequency pulse frequency offset can shift artefacts away from the area of interest. (Figure 1)

### Flow artefact on SSFP images

**Figure 2:**
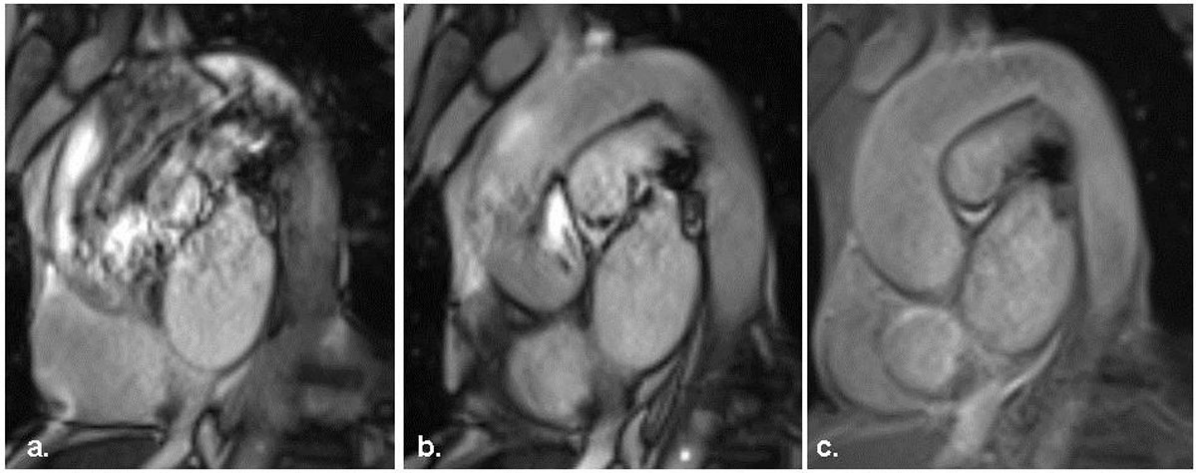
(a) A sagittal oblique still of an SSFP cine of the aorta. Standard shimming applied, significant flow artefact seen. (b) Repeated with targeted cardiac shim, flow artefact is reduced. (c) Switched to a gradient echo sequence, flow artefact removed.

Switching to gradient echo with no flow compensation together with tight shimming reduces the appearance of flow artefacts within vessels. (Figure 2)

### Perfusion imaging

Increased SNR results in improved spatial resolution for single shot acquisition. Reduce phase resolution to decrease TR and maintain single beat acquisition for high heart rates (>115bpm)

### T1-mapping

Ensure proper adjustment of shim and center frequency to minimize off resonance Routinely isocenter. Use large field-of-view (~400mm) and consider motion-corrected or shortened T1-mapping sequence (ShMOLLI) if available, to minimize breathing artefacts

## Conclusions

Good quality CMR images at 3.0T are achievable with adapted scanning techniques and meticulous image optimisation. This improves the diagnostic value of acquired images, minimises scanning time and therefore improves subject experience and throughput in the clinical setting.

## Funding

N/A.

